# Production of Recombinant S1 Protein of Porcine Epidemic Diarrhea Virus in Recombinant CHO Cells for Application in Indirect ELISA

**DOI:** 10.4014/jmb.2506.06050

**Published:** 2025-09-17

**Authors:** Eun-Ji Lee, Sungkyun Kim, Tae-Ho Kim, Na-Yeong Heo, Hyun-Seung Kim, So Hui Ryu, Seung Jin Koo, Hokeun Won, Yeon-Gu Kim

**Affiliations:** 1 Biotherapeutics Translational Research Center, Korea Research Institute of Bioscience and Biotechnology (KRIBB), Daejeon, Republic of Korea; 2 Department of Bioprocess Engineering, KRIBB School of Biotechnology, University of Science and Technology (UST), Daejeon, Republic of Korea; 3 Choong Ang Vaccine Laboratory Co., Ltd. (CAVAC), Daejeon, Republic of Korea

**Keywords:** Porcine epidemic diarrhea virus, spike protein, Fc-fusion protein, recombinant Chinese hamster ovary cells, indirect ELISA

## Abstract

The *N*-terminal S1 subunit of the spike protein (S1 protein) of porcine epidemic diarrhea virus (PEDV) is recognized as a potential diagnostic antigen for detecting anti-PEDV immunoglobulin A (IgA) levels in colostrum, a key indicator for assessing passive immunity against PEDV infection. Given the advantages of producing Fc-fusion proteins in mammalian cells, it is important to investigate how the addition of the Fc region affects the diagnostic performance of the PEDV-S1 protein. In this study, we successfully produced full-length PEDV-S1 protein fused with an Fc region (PEDV-S1-Fc protein) via transient gene expression in recombinant Chinese hamster ovary (rCHO) cells adapted to serum-free suspension culture. The resulting expression showed significantly higher volumetric productivity than previously reported. The aglycosylated form of PEDV-S1-Fc protein was also generated using PNGase F treatment of the purified protein, as well as tunicamycin treatment to inhibit glycosylation during cell culture. Notably, neither the addition of the Fc region nor the removal of *N*-glycans markedly affected the diagnostic function, as demonstrated by indirect ELISA using PEDV-positive and PEDV-negative colostrum samples. Moreover, the diagnostic performance of the PEDV-S1-Fc protein was further validated using total IgA purified from PEDV-positive colostrum. In summary, the full-length Fc-fused PEDV-S1 protein produced in rCHO cell culture exhibited high productivity and purity, making it a promising antigenic candidate for indirect ELISA-based detection of anti-PEDV IgA in colostrum.

## Introduction

Porcine epidemic diarrhea, characterized by severe diarrhea, acute enteritis, and vomiting, leads to high morbidity and mortality in neonatal piglets and is caused by porcine epidemic diarrhea virus (PEDV) [1]. Minimizing economic losses from PEDV outbreaks relies largely on protecting neonatal piglets, as they are significantly more vulnerable to the virus than older pigs [1]. It is well-established that the protection of neonatal piglets depends on maternal secretory immunoglobulin A (IgA) antibodies, with the concentration of PEDV-specific IgA serving as a key indicator for evaluating the level of passive immunity against PEDV infection [2]. Colostrum and milk samples from sows are considered practical for detecting anti-PEDV IgA antibodies; they require less as technical expertise and fewer aseptic conditions than serum samples typically used for ELISA.

PEDV is an enveloped, positive-sense single-stranded RNA virus composed of 16 non-structural proteins and four structural proteins: spike (S), membrane, envelope, and nucleocapsid [3]. The S protein, a trimeric surface glycoprotein composed of an *N*-terminal S1 subunit and a *C*-terminal S2 subunit, has emerged as a promising antigenic candidate for ELISA-based detection of PEDV-specific antibodies due to its ability to induce neutralizing antibodies through vaccination or natural infection [4]. The expression of the PEDV S protein, including both S1 and S2 subunits, has been explored in several systems, such as *Escherichia coli*, yeast, insect, and mammalian cells, for use in diagnostics and vaccine development [5-10]. However, full-length expression of the PEDV-S1 protein in bacterial systems has not yet been achieved, and only truncated forms have been successfully expressed in *E. coli* [6, 10]. Notably, the full-length PEDV-S1 protein has been produced exclusively in mammalian cells, and its expression levels in yeast and insect cells are reported to be 10–100-fold lower than those in mammalian expression systems [8].

Recently, mammalian cell-based recombinant protein production has expanded beyond therapeutic applications to include the generation of various functional proteins, such as diagnostic antigens. Among mammalian cell lines, recombinant Chinese hamster ovary (rCHO) cells adapted to serum-free suspension culture are widely used in the biopharmaceutical industry [11, 12]. Notably, the use of serum-free media in rCHO cell culture offers distinct advantages for diagnostic applications, as it minimizes the risk of contamination by non-specific antibodies present in fetal bovine serum found in serum-containing media. Moreover, rCHO cell-based transient gene expression (TGE) in suspension culture has emerged as a rapid, simple, and cost-effective platform for recombinant protein production, especially compared to adherent culture systems [13]. Despite technological advances in therapeutic protein production using mammalian cells, these improvements have not yet been applied to the PEDV-S1 protein for diagnostic use. Consequently, the effects of enhanced productivity and glycan modulation on the protein’s diagnostic function remain unclear.

In this study, we produced the PEDV-S1 protein in three formats: Fc-fusion, 6 × His-tagged, and aglycosylated using an rCHO cell-based TGE system in serum-free suspension culture. We assessed protein purity and *N*-glycan profiles of the purified PEDV-S1 proteins using size-exclusion high-performance liquid chromatography (HPLC) and anion exchange HPLC. Additionally, we evaluated the diagnostic performance of each protein variant in detecting anti-PEDV IgA antibody levels via ELISA using colostrum samples collected from local farms in the Republic of Korea.

## Materials and Methods

### Construction of PEDV-S1 Protein-Producing Vectors

The S1 domain of PEDV-S1 was obtained from CAVAC (Republic of Korea) and subcloned into the pFuse-hIgG1-Fc1 vector (InvivoGen, USA), resulting in the pFuse-PEDV-S1-hIgG-Fc vector for the production of PEDV-S1 protein with Fc-fusion (PEDV-S1-Fc protein). The amino acid sequence of the PEDV-S1 protein used in this study, which is not available in GenBank, is provided in Table S1. The PEDV-S1 gene fused with a 6 × His-tag was generated by using a reverse primer containing the 6 × His-tag sequence at the C-terminus of the PCR-amplified *PEDV-S1* gene. This gene was subcloned into the pcDNA3.1/Zeo(+) vector (Thermo Fisher Scientific, USA) to create the pcDNA-PEDV-S1-His vector for producing 6 × His-tagged PEDV-S1 protein (PEDV-S1-His protein). Two expression vectors were generated using In-Fusion^®^ Snap Assembly Master Mix (Takara Bio, Japan) as shown in Fig. S1. Both PEDV-S1 protein expression vectors were purified using the EndoFree Plasmid Maxi Kit (Qiagen, Germany) according to the manufacturer’s instructions. Only vectors with an *A*_260_/*A*_280_ >1.8 were used in this study.

### Cell Culture, Maintenance, and TGE

ExpiCHO-S^TM^ cells (Thermo Fisher Scientific) were maintained in serum-free suspension culture mode using ExpiCHO^TM^ Expression Medium (Thermo Fisher Scientific). Cells were cultured in a Climo-shaking CO_2_ incubator (Adolf Kühner, Switzerland) at 37°C, 110 rpm, and 70% humidity. TGE with ExpiCHO-S^TM^ cells was performed using the two PEDV-S1 protein expression vectors, following the manufacturer’s protocol as previously described [14]. Suspension cells cultured in ExpiCHO^TM^ Expression Medium were harvested at mid-exponential phase by centrifugation and resuspended in fresh medium at a density of 6 × 10^6^ cells/ml in Corning^®^ Erlenmeyer cell culture flasks (Corning, USA). For transfection, plasmid DNA was diluted in OptiPRO^TM^ SFM (Thermo Fisher Scientific), and ExpiFectamine^TM^ CHO Reagent (Thermo Fisher Scientific) was separately diluted in OptiPRO^TM^ SFM. The two solutions were then combined to form the transfection complex, which was immediately added to the cells. At 20 h post-transfection, ExpiCHO^TM^ Enhancer (Thermo Fisher Scientific) and ExpiCHO^TM^ Feed (Thermo Fisher Scientific) were added to the culture to enhance protein expression. For the production of aglycosylated PEDV-S1 protein, tunicamycin (1 μg/ml; Sigma-Aldrich, USA) was added to the culture 48 h post-transfection [15].

### Cell Concentration, Viability, and PEDV-S1 Protein Assay

Cell concentration and viability were assessed using a Cedex HiRes analyzer (Roche Diagnostics, Switzerland) with the trypan blue dye exclusion method. The concentration of secreted PEDV-S1-Fc protein in the culture supernatant was quantified using a Cedex Bio analyzer (Roche Diagnostics) according to the manufacturer’s instructions. Evaluation of PEDV-S1-His protein in the culture supernatant was performed via Western blot analysis using an anti-His horseradish peroxidase (HRP)-conjugated antibody (Santa Cruz Biotechnology, USA).

### Purification of PEDV-S1 Protein

PEDV-S1-Fc and PEDV-S1-His proteins were purified from culture supernatants using protein A chromatography with MabSelect^TM^ PrismA (Cytiva, UK) and immobilized metal affinity chromatography with Ni Sepharose^TM^ Excel (Cytiva), respectively, on a liquid chromatography system (ÄKTA pure 25 L; Cytiva). The purified proteins were separated on a Bolt^TM^ 4%–12% Bis–Tris gel (Thermo Fisher Scientific), and the gels were stained with InstantBlue^TM^ (Abcam, UK).

### Size-Exclusion Chromatography (SEC) Analysis

Purified PEDV-S1 protein was separated using an AdvanceBio SEC 300 Å column (2.7 μm, 4.6 mm × 300 mm; Agilent Technologies, USA) connected to a UHPLC system (1290 Infinity II Bio LC system; Agilent Technologies), following the manufacturer’s protocol. The mobile phase consisted of 150 mM sodium phosphate (pH 7.0), and the signal was monitored by UV absorbance at 220 nm.

### Sialylated *N*-Linked Glycan Structure Analysis of PEDV-S1 Protein

Sialylated *N*-linked glycans of the purified PEDV-S1 protein were analyzed as described previously [16]. The glycans were released by PNGase F (Roche Diagnostics) treatment and labeled with 2-aminobenzamide (2-AB; Sigma-Aldrich), a fluorescent reagent. The labeled *N*-glycans were separated on an Agilent Bio SAX column (5 μm, 2.1 mm × 250 mm; Agilent Technologies) connected to a UHPLC system (1290 Infinity II Bio LC system; Agilent Technologies) and detected using a fluorescence detector.

### ELISA with PEDV-S1 Protein for PEDV-Positive/Negative Colostrum

The purified PEDV-S1 proteins were evaluated using a conventional ELISA with PEDV-positive and PEDV-negative colostrum samples obtained from CAVAC, as previously described [16]. Briefly, 96-well plates (Corning) were coated with either purified PEDV-S1-Fc (100 ng/well) or PEDV-S1-His protein (75 ng/well) to ensure equal molar quantities, followed by blocking with a standard blocking buffer. The plates were incubated with PEDV-positive or negative colostrum samples diluted 1:300. Total IgA was isolated using HiTrap^TM^ Protein L resin (Cytiva) and quantified using the IgA Pig ELISA kit (Abcam). Total IgA purified from PEDV-positive colostrum was tested at various concentrations. Following sample incubation, HRP-conjugated goat anti-pig IgA secondary antibody (Thermo Fisher Scientific) was added. The enzymatic reaction was visualized using BioFX^TM^ TMB substrate (Surmodics, USA).

### Virus Neutralization (VN) Assay for PEDV

The VN assay for PEDV was performed using Vero cells, as previously described [10]. Briefly, colostrum samples were serially diluted and mixed with an equal volume of PEDV at 2 × 10^3^ TCID_50_/ml. After a 1 h incubation to allow neutralization, each mixture was added to confluent Vero cells in a 96-well culture plate (Corning) and incubated for 1 h to permit viral adsorption. The inoculum was then removed, cells were washed with sterile phosphate-buffered saline, and virus propagation medium was added to each well. Following incubation in a 5% CO_2_ atmosphere for 3–5 days, wells were examined for cytopathic effects (CPE). The neutralizing antibody titer was defined as the reciprocal of the highest colostrum dilution that completely inhibited CPE.

## Results and Discussion

### Effect of Adding the Fc Region to the PEDV-S1 Protein on Diagnostic Function

Recombinant protein production in the form of Fc fusions using mammalian cell-based expression systems is commonly employed due to advantages in production efficiency, high-yield purification, and ease of quantification [17]. However, as the Fc region has a relatively large molecular weight compared to commonly used peptide tags such as the 6 × His-tag, FLAG-tag, and c-Myc tag [18], it may impede the diagnostic function of Fc-fusion proteins. This limitation may also apply to the PEDV-S1 protein fused with Fc, which was successfully expressed in HEK293 cells in a previous study, although its diagnostic function was not validated [5]. To evaluate the diagnostic efficiency of the Fc-fused PEDV-S1 protein (PEDV-S1-Fc protein) and the 6 × His-tagged PEDV-S1 protein (PEDV-S1-His protein), two PEDV-S1 protein expression vectors were constructed and expressed using the rCHO cell-based TGE system. The cell culture supernatants were harvested at over 90% cell viability to minimize the impact of proteases released from membrane-disrupted cells and then purified using protein A chromatography and immobilized metal affinity chromatography.

**Fig. 1:**
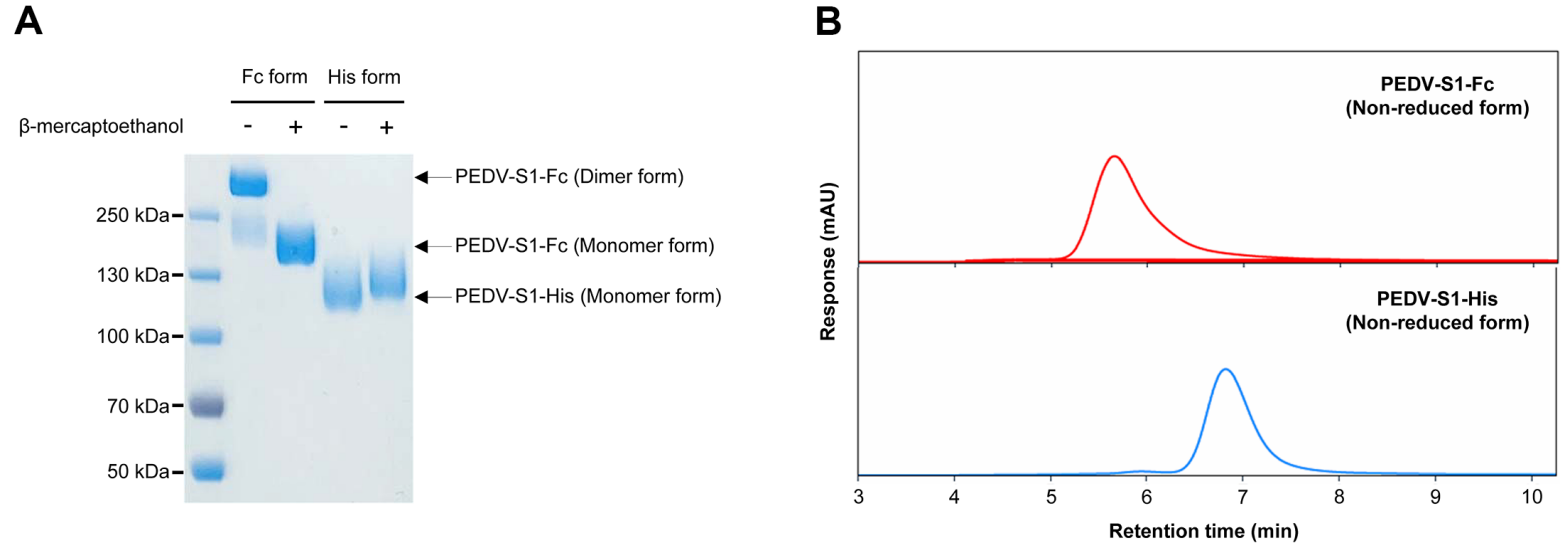
Expression pattern and purity of purified PEDV-S1 proteins with Fc-fusion (PEDV-S1-Fc) and 6 × His-tag (PEDV-S1-His) produced using the rCHO cell-based TGE system. (**A**) SDS-PAGE analysis of PEDV-S1-Fc and PEDV-S1-His proteins under reducing (+) and non-reducing (‒) conditions using β-mercaptoethantol. Protein expression was performed via the TGE system, and the resulting cell culture supernatants were purified using protein A affinity and immobilized metal chelate affinity chromatography, respectively. Equal amounts of purified proteins were loaded under reducing and non-reducing conditions and stained with Coomassie brilliant blue. (**B**) Size exclusion chromatography (SEC) analysis of non-reduced PEDV-S1-Fc and PEDV-S1-His proteins. Equal amounts of the purified proteins were loaded onto an SEC-HPLC column, and UV absorbance was monitored.

Fig. 1 shows the expression profile and purity of PEDV-S1 proteins with Fc-fusion and 6 × His-tag, produced in rCHO cells, as assessed by SDS-PAGE and SEC analyses. The product titers of PEDV-S1-Fc and PEDV-S1-His proteins produced using the rCHO cell-based TGE system under serum-free suspension culture conditions were 217.5 ± 12.1 mg/l and 101.5 ± 8.1 mg/l on day 10, respectively. These were significantly higher than the yield (30 mg/l) obtained from HEK293T cells for PEDV-S1-His protein [8]. As PEDV-S1-Fc proteins can form dimers via disulfide bonds in the Fc region, the PEDV-S1-Fc protein was clearly observed as a 150 kDa band under reducing conditions and a 300 kDa band under non-reducing conditions (Fig. 1A). In contrast, the PEDV-S1 protein itself does not dimerize, as shown by the unchanged mobility of the PEDV-S1-His protein regardless of the presence or absence of a reducing agent (Fig. 1A). The purity of PEDV-S1-Fc and PEDV-S1-His proteins was 96.8% ± 2.1% and 95.5% ± 1.5%, respectively, as determined by SEC analysis, remarkable purity levels considering that only a single purification step was used (Fig. 1B). Additionally, highly purified PEDV-S1-Fc and PEDV-S1-His proteins were further verified by Western blot analysis to confirm the presence of Fc region and 6 × His tag, respectively (Fig. S2). In summary, full-length PEDV-S1 protein production in rCHO cell-based TGE with serum-free suspension culture is highly efficient in terms of both yield and purification, regardless of the type of fusion tag used.

**Fig. 2:**
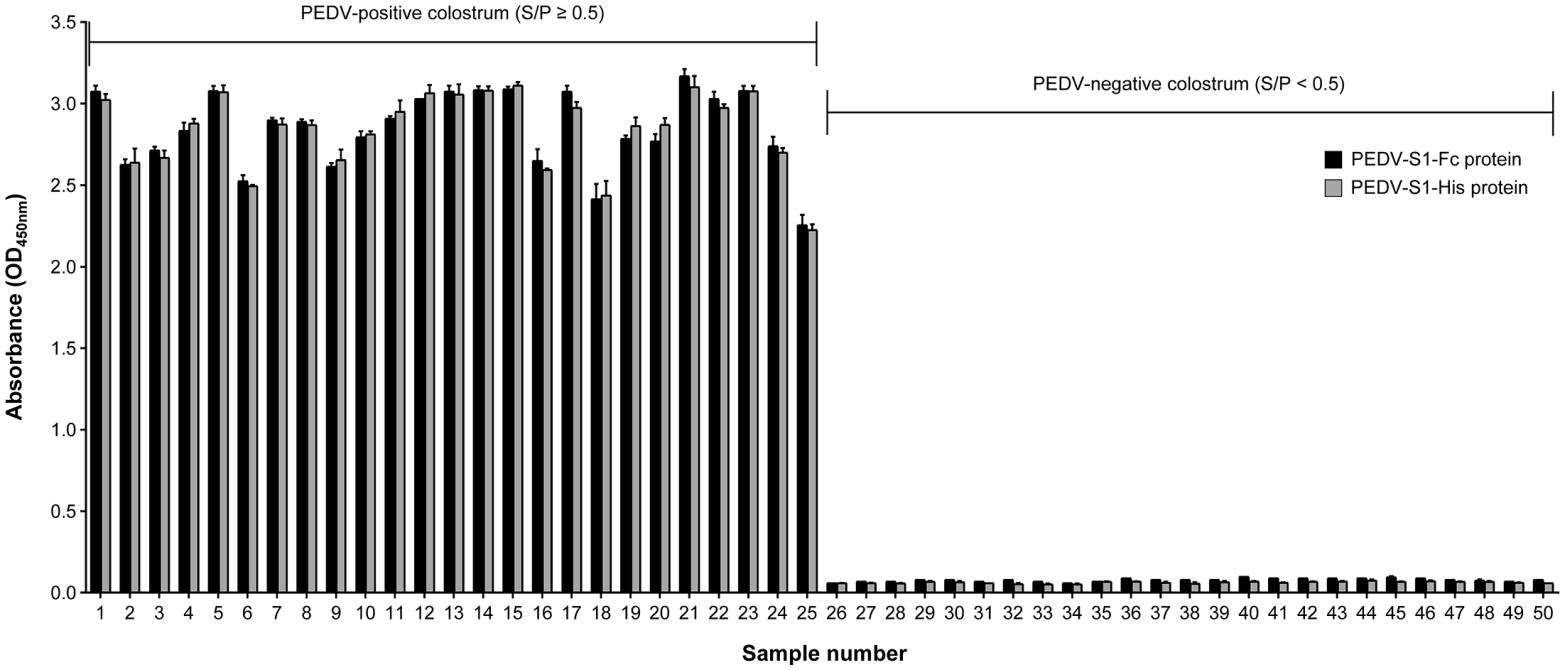
Binding affinity assay of anti-PEDV IgA antibodies in colostrum using PEDV-S1-Fc (black) and PEDV-S1-His (gray) proteins as antigens in an indirect ELISA. A total of 50 colostrum samples were tested and categorized into two groups based on the virus neutralization (VN) assay: a positive group (S/P ratio ≥0.5) and a negative group (S/P ratio <0.5). Error bars indicate standard deviations calculated from technical triplicates.

Fig. 2 illustrates the detection pattern of anti-PEDV IgA antibodies using PEDV-S1 proteins with Fc-fusion and 6 × His-tag as antigens, as determined by an indirect ELISA with PEDV-positive and -negative colostrum samples. Among the colostrum samples collected from farms in the Republic of Korea, PEDV-positive (*n* = 25) and PEDV-negative (*n* = 25) samples were confirmed using the VN assay. Colostrum were further evaluated using the IDEXX PEDV IgA Antibody Test Kit, and classified as PEDV-positive when the sample-to-positive (S/P) ratio was ≥ 0.5 and as PEDV-negative when the S/P ratio was < 0.5. As expected, the OD_450 nm_ values from the ELISA for anti-PEDV IgA levels were significantly higher in PEDV-positive colostrum samples compared to PEDV-negative samples, regardless of whether the PEDV-S1-Fc or PEDV-S1-His protein was used as the antigen. Notably, no significant difference in diagnostic performance was observed between the two antigen types. These findings suggest that the PEDV-S1-Fc protein is a viable diagnostic antigen candidate for establishing an indirect ELISA to detect anti-PEDV IgA antibodies in colostrum samples.

### Effect of *N*-Glycan Removal from PEDV-S1 Protein on Diagnostic Function

The PEDV-S1 protein produced in mammalian cells is known to undergo *N*-linked glycosylation but not O-linked glycosylation, as demonstrated by enzymatic treatment with PNGase F and O-glycosidase [8]. To confirm the presence of *N*-linked glycans in PEDV-S1 protein expressed in rCHO cells, sialylated *N*-linked glycans released from purified PEDV-S1 proteins were analyzed using an anion exchange HPLC column. The labeled *N*-linked glycans were categorized into five groups - neutral, mono-, di-, tri-, and tetra-sialylated—based on their charge and quantified by integrating the peak areas of each group in the HPLC analysis.

**Fig. 3:**
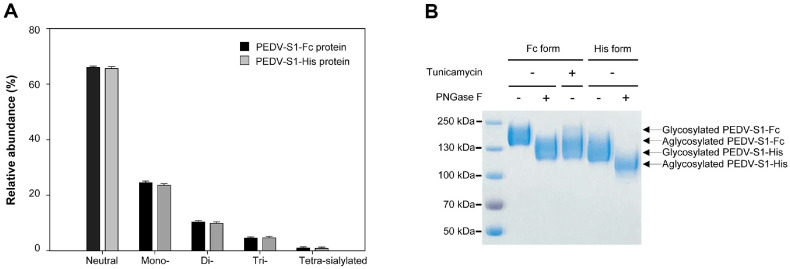
Glycosylation analysis of purified PEDV-S1-Fc and PEDV-S1-His proteins produced using the rCHO cell-based TGE system. (**A**) Anion exchange HPLC analysis of sialylated *N*-linked glycans derived from the purified PEDVS1 proteins. The relative abundances of neutral, mono-, di-, tri-, and tetra-sialylated *N*-linked glycans were measured for PEDV-S1-Fc (black) and PEDV-S1-His (gray) proteins. Error bars represent standard deviations calculated from technical triplicates. (**B**) SDS-PAGE analysis of glycosylated and aglycosylated forms of PEDV-S1-Fc and PEDV-S1-His proteins. Equal amounts of protein were loaded under reducing conditions, and the gel was stained with Coomassie brilliant blue.

Fig. 3A presents the distribution of the five *N*-linked glycan types in PEDV-S1-Fc and PEDV-S1-His proteins produced in rCHO cells. As anticipated, the glycan pattern of the purified PEDV-S1-His protein closely resembled that of the PEDV-S1-Fc protein. Additionally, NetNGlyc 1.0, a predictive tool for *N*-glycosylation sites, confirmed that PEDV-S1 contains *N*-linked glycosylation sites (Fig. S3). Given the confirmed presence of *N*-linked glycans in PEDV-S1 protein produced in rCHO cells, it is important to assess whether glycan formation impacts its diagnostic performance as an antigen in indirect ELISA for detecting anti-PEDV IgA antibodies. As a preliminary step in this evaluation, an aglycosylated form of PEDV-S1 protein was generated through PNGase F treatment of purified PEDV-S1-Fc protein and by using tunicamycin-supplemented TGE to inhibit glycosylation, respectively [8, 19].

Fig. 3B displays the expression patterns of PEDV-S1-Fc and PEDV-S1-His proteins with and without *N*-linked glycans, as analyzed by SDS-PAGE. Typically, the SDS-PAGE bands of aglycosylated forms meaning non-glycosylated versions of glycoproteins shift from higher to lower molecular weight following the removal of *N*-linked glycans [20]. Aglycosylated forms of both PEDV-S1-Fc and PEDV-S1-His proteins were successfully generated by treating their glycosylated counterparts with PNGase F, an enzyme that hydrolyzes all *N*-linked glycosylation sites. Additionally, PEDV-S1-Fc protein lacking *N*-linked glycans was produced using the rCHO cell-based TGE system supplemented with tunicamycin, a commonly used glycosylation inhibitor in mammalian cells. Subsequently, an indirect ELISA was conducted to evaluate the diagnostic performance of PEDV-S1-Fc proteins with and without *N*-linked glycans generated either by enzymatic deglycosylation or by modified culture conditions for detecting anti-PEDV IgA antibodies.

**Fig. 4:**
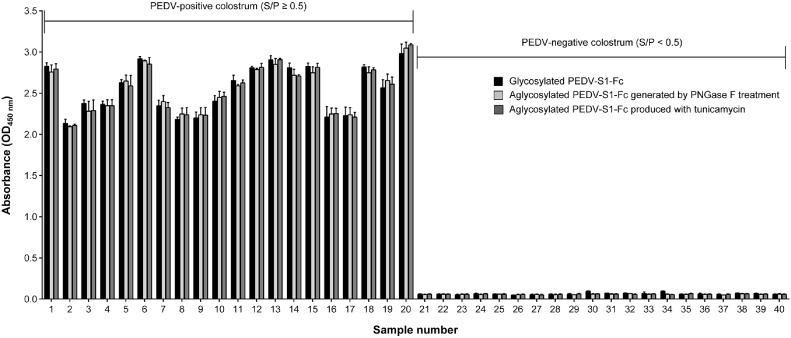
Binding affinity assay for anti-PEDV IgA antibodies in colostrum using glycosylated (black) and aglycosylated (light gray and dark gray) PEDV-S1-Fc proteins as antigens in an indirect ELISA. Samples with an S/P ratio ≥0.5 were classified as PEDV-positive, while those with an S/P ratio <0.5 were considered negative. Error bars indicate standard deviations based on technical triplicates.

Fig. 4 presents the detection pattern of anti-PEDV IgA antibodies by indirect ELISA using glycosylated and aglycosylated forms of PEDV-S1-Fc protein. PEDV-positive and -negative colostrum samples were classified via VN assay, as described in Fig. 2. Notably, the absence of *N*-linked glycans in PEDV-S1-Fc protein did not significantly impact its diagnostic function as an antigen for detecting anti-PEDV IgA levels. A similar outcome was observed for PEDV-S1-His protein, irrespective of glycosylation status (data not shown). HEK293 cells, alongside rCHO cells, are widely used mammalian hosts for recombinant protein production, but they tend to generate glycoproteins with more complex glycan structures than those from rCHO cells [21]. Nevertheless, PEDV-S1-Fc protein produced in HEK293 cells using the TGE system exhibited no detectable difference in diagnostic functionality compared to that produced in rCHO cells (data not shown). Collectively, these results suggest that modulation of *N*-glycan formation in PEDV-S1 protein expressed in mammalian cells does not influence its utility as a diagnostic antigen in ELISA-based assays.

### In-House ELISA Using PEDV-S1-Fc Protein for Purified Total IgA from PEDV-Positive Colostrum

To determine whether the PEDV-S1-Fc protein specifically binds to PEDV-specific IgA in colostrum, total IgA was purified from PEDV-positive colostrum using Protein L chromatography. As a control, total IgA was also purified from PEDV-negative colostrum. The purified total IgA was quantified using a commercially available pig-specific IgA detection kit.

**Fig. 5:**
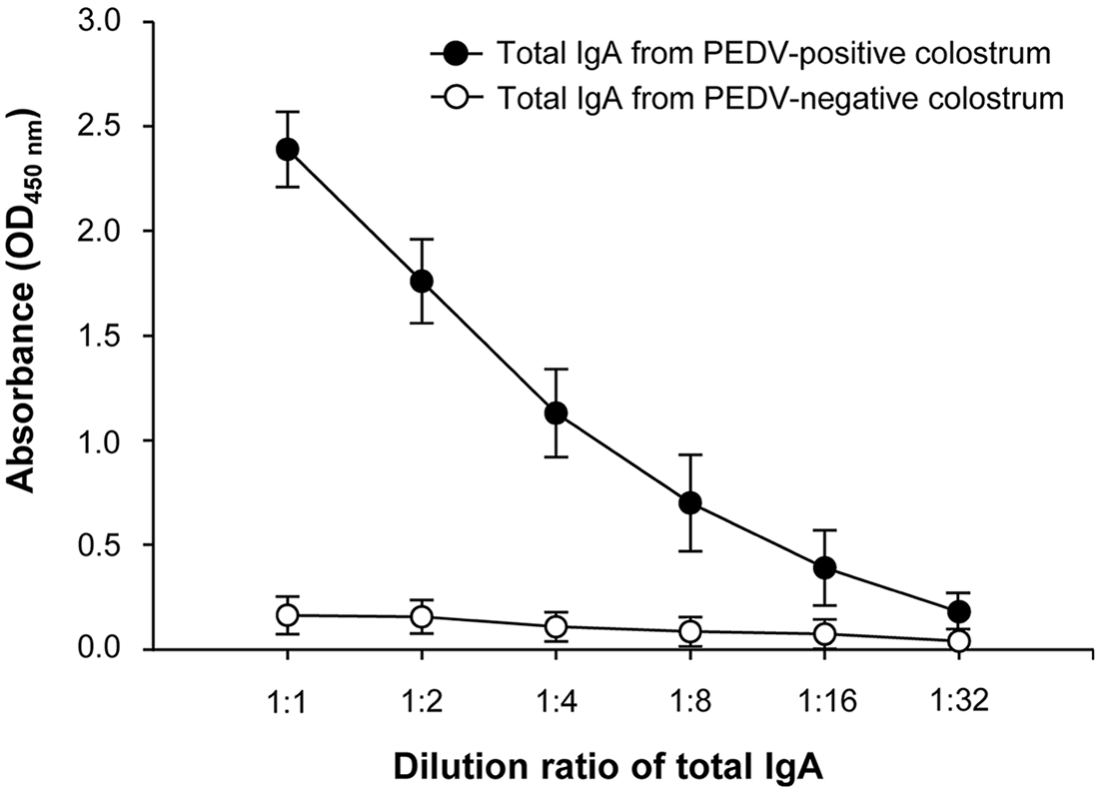
Comparison of binding affinity for anti-PEDV IgA antibodies in total IgA samples purified from PEDV-positive and PEDV-negative colostrum using PEDV-S1-Fc proteins as antigens in an indirect ELISA. Quantified total IgA samples were serially diluted twofold, with a maximum dilution of 1:32. Error bars represent standard deviations calculated from technical triplicates.

Fig. 5 illustrates the detection pattern of anti-PEDV IgA antibodies using PEDV-S1-Fc protein as the antigen in an indirect ELISA, employing purified total IgA from PEDV-positive and -negative colostrum. First, we confirmed the presence of PEDV-specific IgA in the total IgA purified from PEDV-positive and -negative colostrum using the IDEXX PEDV IgA Antibody Test Kit (data not shown). The quantified total IgA samples were subjected to two-fold serial dilutions, with a maximum dilution ratio of 1:32. The ELISA OD_450 nm_ values for anti-PEDV IgA levels demonstrated a strong dose-dependent response in the total IgA sample from PEDV-positive colostrum. As expected, there was no significant change in OD_450 nm_ values regardless of dilution concentration in total IgA samples from PEDV-negative colostrum. Collectively, these results demonstrate that PEDV-S1-Fc protein produced in rCHO cells can specifically detect PEDV-specific IgA in purified total IgA from PEDV-positive colostrum. Furthermore, purified total IgA can serve as a reliable quality control material in an indirect ELISA for detecting PEDV-specific IgA antibodies.

The production of Fc-fusion proteins in mammalian cells offers advantages in high-yield production efficiency due to enhanced secretion capacity, simplified purification through protein A chromatography, and convenient quantification using high-quality analytical instrumentation [17]. In this context, Fc-fusion protein-based ELISA platforms provide a cost-effective and high-quality approach for developing serological diagnostic kits [22]. Despite these advantages, the full-length PEDV-S1 protein fused with Fc, produced in mammalian cells, has not yet been employed as an antigen in the indirect ELISA method for detecting anti-PEDV IgA antibody levels. Gerber *et al*. (2014) previously produced the full-length PEDV-S1-Fc protein in HEK293F cells; however, the diagnostic application involved PEDV-S1 protein with the Fc region removed. Thus, it is important to determine whether the full-length PEDV-S1-Fc protein can serve effectively in an indirect ELISA method for PEDV-IgA antibody detection. In this study, we successfully produced the full-length PEDV-S1-Fc protein using an rCHO cell-based TGE system under serum-free suspension culture conditions (Fig. 1), achieving a production level 7.25-fold higher than that of the PEDV-S1-His protein previously reported in HEK293T cells [8]. Furthermore, we showed that addition of a larger Fc region to the PEDV-S1 protein compared to the 6 × His tag did not compromise the diagnostic performance for detecting anti-PEDV IgA antibody in colostrum samples (Figs. 2 and 5).

The *N*-glycosylation pattern of therapeutic glycoproteins produced in mammalian cells significantly influences their biological functions, including efficacy, stability, and antigen binding [23]. These proteins, including Fc-fusion glycoproteins, typically feature more complex tetra-antennary glycan structures compared to the bi-antennary glycans found in the Fc region of canonical antibodies [24, 25]. Therefore, the production of therapeutic glycoproteins in rCHO cells requires careful optimization of the cell culture process, with particular attention to maintaining appropriate *N*-glycosylation patterns [26, 27]. In contrast to the critical role of *N*-glycosylation in therapeutic glycoproteins, we demonstrated that removal of *N*-glycans from the PEDV-S1-Fc protein did not compromise its diagnostic performance (Fig. 4). This indicates that strict maintenance of *N*-glycosylation patterns is not essential for diagnostic antigens, offering greater flexibility in the selection of mammalian host cells. Unlike therapeutic protein production, where rCHO cells are often preferred despite their high cost, diagnostic antigen production can be achieved using a range of mammalian cell lines, thereby reducing costs without sacrificing assay quality. These findings suggest that various mammalian cell lines, excluding expensive commercial options, can effectively serve as host cells for PEDV-S1 protein production.

In conclusion, we successfully produced full-length PEDV-S1 protein with Fc-fusion in rCHO cells under serum-free suspension culture conditions. Furthermore, we confirmed that the Fc-fusion form of the PEDV-S1 protein can specifically detect anti-PEDV IgA antibodies through the indirect ELISA method, using PEDV-positive/negative colostrum and purified PEDV IgA derived from PEDV-positive colostrum.

## Supplemental Materials

Supplementary data for this paper are available on-line only at http://jmb.or.kr.
