# Supplementary material for: Production of Recombinant S1 Protein of Porcine Epidemic Diarrhea Virus in Recombinant CHO Cells for Application in Indirect ELISA
Source: J Microbiol Biotechnol. 2025 Sep 17;35:e2506050. doi: 10.4014/jmb.2506.06050 (PMC12463560; doi:10.4014/jmb.2506.06050)
Supplement: Supplementary file 1 [file jmb-35-e2506050-supple.pdf]

J. Microbiol. Biotechnol. 2025. 35: e2506050

https://doi.org/10.4014/jmb.2506.06050

Supplementary Table and Figures

Production of Recombinant S1 Protein of Porcine Epidemic Diarrhea Virus in Recombinant CHO Cells for Application in Indirect ELISA

Eun-Ji Lee[1,2a], Sungkyun Kim[3,a], Tae-Ho Kim[1], Na-Yeong Heo[1,2], Hyun-Seung Kim[1,2], So Hui Ryu[1], Seung Jin Koo[1,2], Hokeun Won[3,*], Yeon-Gu Kim[1,2*]

[1]*Biotherapeutics Translational Research Center, Korea Research Institute of Bioscience and Biotechnology (KRIBB), 125 Gwahak-ro, Yuseong-gu, Daejeon, Korea*

[2]*Department of Bioprocess Engineering, KRIBB School of Biotechnology, University of Science and Technology (UST), 217 Gajeong-ro, Yuseong-gu, Daejeon, Korea*

[3]*Choong Ang Vaccine Laboratory Co., Ltd. (CAVAC), 1476-37 Yuseong-daero, Yuseong-gu, Daejeon, Korea*

**Co-correspondence to: Dr. Hokeun Won*

E-mail: hokeun@cavac.co.kr

**Co-correspondence to: Dr. Yeon-Gu Kim*

E-mail: ygkim@kribb.re.kr

[a]Eun-Ji Lee and Sungkyun Kim contributed equally to this work.

Table S1. Amino acid sequence of PEDV-S1 protein used in this study.

Name	Amino acid sequence
PEDV-S1 protein	MRSLTYFWLFLPVLSTLSLPQDVTRCSANTNFRRFFSKFN<br>VQAPAVVVLGGYLPIGENQGVNSTWYCAGQHPTASGVH<br>GIFVSHIRGGHGFEIGISQEPFDPSGYQLYLHKATNGNTNA<br>TARLRICQFPSIKTLGPTANNDVTTGRNCLFNKAIPAHMSE<br>HSVVGITWDNDRVTVFSDKIYYFYFKNDWSRVATKCYNS<br>GGCAMQYVYEPTYYMLNVTSAGEDGISYQPCTANCIGYA<br>ANVFATEPNGHIPEGFSFNNWFLLSNDSTLVHGKVVSNQP<br>LLVNCLLAIPKIYGLGQFFSFNQTIDGVCNGAAVQRAPEA<br>LRFNINDTSVILAEGSIVLHTALGTNFSFVCSNSSNPHLATF<br>AMPLGATQVPYYCFLKVDTYNSTVYKFLAVLPPTVREIVI<br>TKYGDVYVNGFEYLHLGLLDAVTINFTGHGTDDDVSGF<br>WTIASTNFVDALIEVQGTAIQRILYCDDPVSQLKCSQVAF<br>DLDDGFYPISSRNLLSHEQPISFVTLPSFNDHSFVNITVSAS<br>FGGHSGANLIASDTTINGFSSFCVDTRQFTISLFYNVTNSY<br>GYVSKSQDSNCPFTLQSVNDYLSFSKFCVSTSLLASACTID<br>LFGYPEFGSGVKFTSLYFQFTKGELITGTPKPLEGVTDVSF<br>MTLDVCTKYTIYGFKGEGIITLTNSSFLAGVYYTSDSGQLL<br>AFKNVTSGAVYSVTPCSFSEQAAYVDDDIVGVISSLSSSTF<br>NSTRELPGFFYHSNDGSNCTEPVLVYSNIGVCKSGSIGYVP<br>SQSGQVKIAPTVTGNISIPTNFSMSI

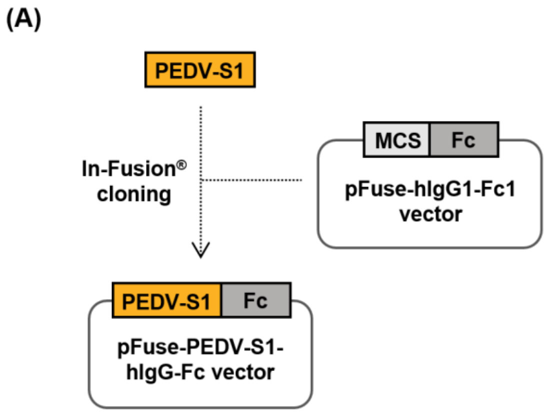

Fig. S1. Schematic diagram of the cloning strategy for the construction of PEDV-S1 protein-producing vectors. (A) The coding region of the *PEDV-S1* gene was inserted into the pFuse-hIgG1-Fc1 vector by In-Fusion® cloning method to generate the pFuse-PEDV-S1-hIgG-Fc vector. This construct enables expression of the PEDV-S1 domain fused to the human IgG1 Fc region. **(B)** The *PEDV-S1* gene, PCR-amplified with a *C*-terminal 6 × His-tag, was inserted into the pcDNA3.1/Zeo(+) vector using the same cloning method to generate the pcDNA-PEDV-S1-His vector, which drives expression of the His-tagged PEDV-S1 protein.

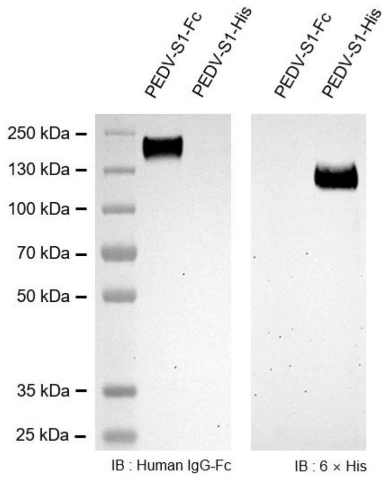

Fig. S2. Western blot analysis of purified PEDV-S1 proteins, each containing either an Fc region or 6 × His-tag. PEDV-S1-Fc and PEDV-S1-His proteins were produced in CHO cells and purified from culture supernatants by protein A chromatography. Equal amounts (1 μg) of each purified protein were electrophoresed on a Bolt^{TM} 4–12% Bis–Tris gel under reducing conditions and transferred onto nitrocellulose membranes. For detection, the following antibodies were used: goat anti-human IgG-Fc fragment antibody (Bethyl Laboratories, USA) and mouse anti-His-tag antibody (Santa Cruz Biotechnology, USA).

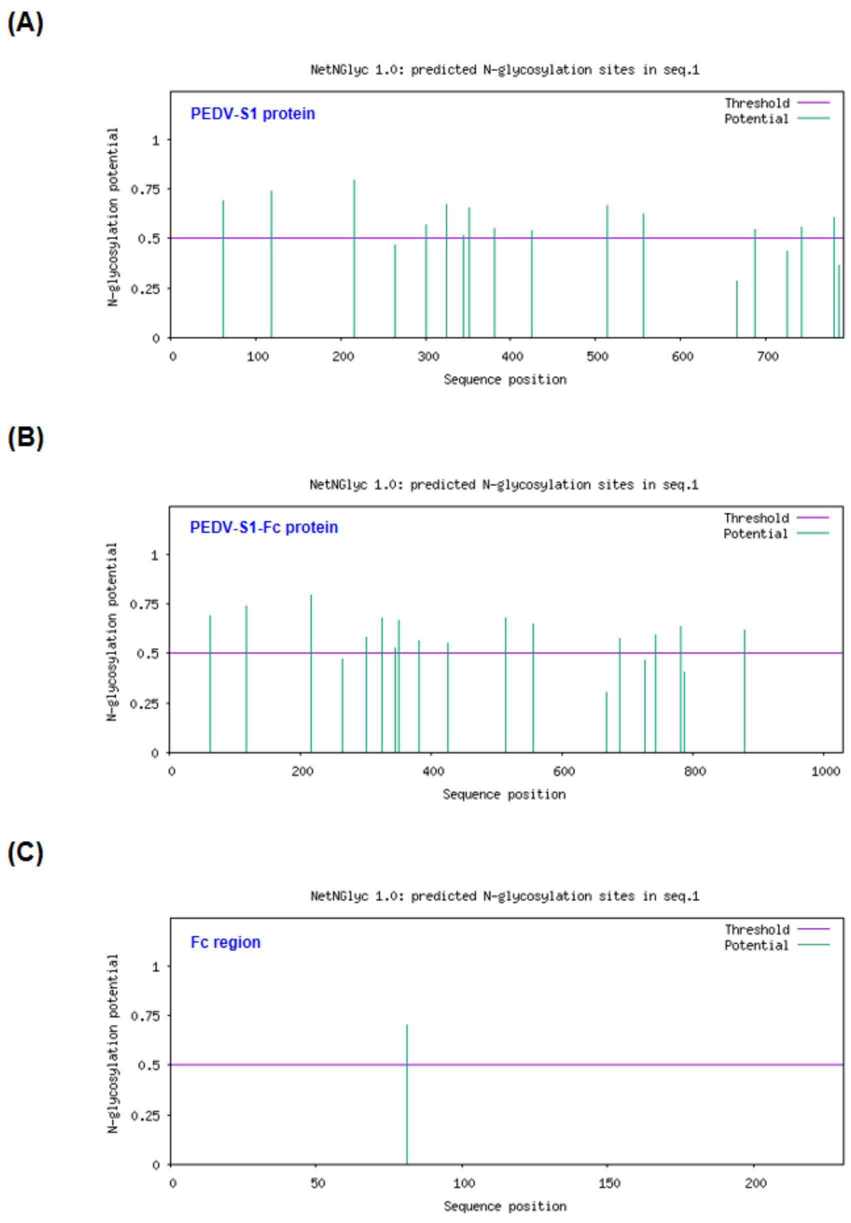

Fig. S3. Prediction of *N*-linked glycosylation sites using the NetNGlyc 1.0 program. The PEDV-S1 protein **(A)** PEDV-S1-Fc fusion protein **(B)** and Fc region **(C)** were analyzed for potential *N*-linked glycosylation sites. Green bars indicate predicted glycosylation potentials exceeding the threshold (purple line).
